# Primary characterization of the immune response in pigs infected with *Trichinella spiralis*

**DOI:** 10.1186/s13567-020-0741-0

**Published:** 2020-02-21

**Authors:** Nan Wang, Xue Bai, Bin Tang, Yong Yang, Xuelin Wang, Hongfei Zhu, Xuenong Luo, Hongbin Yan, Hong Jia, Mingyuan Liu, Xiaolei Liu

**Affiliations:** 1 Key Laboratory of Zoonosis Research, Ministry of Education, Institute of Zoonosis/College of Veterinary Medicine, Jilin University, Changchun, 130000 China; 2 Institute of Animal Sciences, Chinese Academy of Agricultural Sciences, Beijing, 100193 China; 3 State Key Laboratory of Veterinary Etiological Biology, Key Laboratory of Veterinary Parasitology of Gansu Province, Lanzhou Veterinary Research Institute, Chinese Academy of Agricultural Sciences, Lanzhou, 730046 China

## Abstract

Trichinellosis, which is caused by *Trichinella spiralis* (*T. spiralis*), is a serious zoonosis. Pigs play an important role in the transmission of human trichinellosis. Characterizing the immune response to *T. spiralis* infection is key to elucidating host–parasite interactions. However, most studies on the immune response to *T. spiralis* infection have employed murine models. In this study, we investigated the immune response to *T. spiralis* infection in pigs. The results showed that the average numbers of larvae per gram (lpg) for the 100-muscle larvae (ML), 1000-ML, and 10 000-ML groups were 1.502, 35.947, and 398.811, respectively. The percentages of CD3+ T cells, B cells, CD4+ T cells, Treg cells, and Th17 cells were elevated in the infection groups compared to the control animals. In contrast, CD8+ T cell percentages were reduced after infection in the low-dose group. The number of neutrophils was increased at 3–17 days post-infection (dpi). Th1 cytokine IL-2 levels were significantly decreased at 7 dpi, and Th2 cytokine IL-4 levels were significantly elevated at 3 dpi. Treg cytokine IL-10 levels were significantly elevated between 7 dpi and 30 dpi. Th17 cytokine IL-17A levels were significantly increased beginning at 11 dpi. These results confirmed that pigs infected with *T. spiralis* predominantly induced Th2 and Treg immune responses, which suppress the Th1 immune responses. This study provides novel insights into the immune response of pigs infected with *T. spiralis*.

## Introduction

Trichinellosis is a frequently occurring zoonosis that leads to significant economic loss in pork production. This disease is distributed worldwide and has a notable impact on human health, with approximately 10 000 cases occurring each year [1]. The main causative agent of trichinellosis, *Trichinella spiralis* (*T. spiralis*), is usually undetected in pig carcasses due to the lack of macroscopically visible alterations, and pigs play an important role in the transmission of human trichinellosis [2–4]. Humans become infected via consumption of inadequately cooked or raw meat (usually pork) containing infective *T. spiralis* larvae [5–7]. After ingestion, infective muscle larvae (ML) exhibit 4 molting events in the intestinal epithelium [8]. Then, the worms develop into adult worms (Ad) and released newborn larvae (NBL), and NBL finally penetrate into the muscle cells, where they grow, transforming the host cells into nurse cells, and develop into ML [5, 9]. To resist the invasion of parasites, the innate and adaptive immune responses of the host are activated and undergo the process of expulsion. In human trichinellosis, the cellular immune response produces a mixed Th1/Th2 immune response with Th2 predominance during the chronic stage [10]. A type 2 cytokine pattern was produced by peripheral blood mononuclear cells (PBMC) of the patient, and the share of CD8+ lymphocytes in the PBMC composition was increased [11]. In rodent models, a stable Th2 immune response is maintained during infection with *T. spiralis* after a short Th1 immune response [12]. However, the cellular immune response in pigs infected with *T. spiralis* is not well known.

The immune response and gene expression in the host and parasite metabolism are switched on at different developmental stages [9, 13, 14]. The parasite can evade the host immune defenses to survive through the cuticle and secretory proteins that regulate the immune response and form nurse cells [13, 15, 16]. A previous study showed that *T. spiralis* infection modulates several signaling pathways in infected animals such that the worms can persistently exist for several years within the host [13]. The regulatory immune response of hosts has a positive effect on the survival of the parasite. A subset of T lymphocytes, the CD4+ T cells, plays a key role in parasitic infection and can polarize into Th1, Th2, Treg, and Th17 cells upon antigenic stimulation. *T. spiralis* and its secretory products can suppress inflammatory responses and induce Th2-type immune responses, as determined by the elevated Th2-associated cytokine levels observed in infected animals [17, 18]. The Th1 phenotype immune response is induced during the intestinal stage and predominantly induces a Th2-type immune response during the ML phase [19]. The role of the Th1-type immune response contributes to parasites elimination [20]. Th2-type immune responses may help alleviating tissue damage and strengthen tissue repair [21]. Treg-type immune responses play a key role in the anti-inflammatory effects of helminth infection and exist in the ML phase [22, 23]. The percentage of Th17 cells is significantly higher in the immunopathology of human schistosomiasis [24]. Th17 cells produce the proinflammatory cytokine IL-17, which plays a role in intestinal inflammation [25]. IL-17 levels are increased during the acute phase of *T. spiralis* infection and are associated with jejunal muscle contractility in mice [26]. In summary, Th1, Th2, Treg, and Th17 cells play important roles in the infection of *T. spiralis*. Therefore, we studied the dynamics of T lymphocytes in pigs infected with *T. spiralis*.

B cells are a major component of the systemic immune response in hosts. B cells play a key role in parasite clearance by producing antibodies [27]. Meanwhile, B cells are important antigen-presenting cells (APCs) that can induce Treg cell production [28]. Neutrophils are essential components in the innate immune responses of the host and play important roles in local immunity during infection by promoting inflammation and fighting infections [29]. Neutrophils can regulate adaptive immune responses by inhibiting or promoting T cell proliferation, depending on activation status [30, 31].

Previous studies have frequently used mice as animal models in research investigating the immune response to trichinellosis, although *T. spiralis* was isolated from pigs [32, 33]. To better understand the whole process of immune responses in pigs infected with *T. spiralis*, this study was performed to analyze the dynamics of T cells, B cells, neutrophils, and cytokines associated with Th1, Th2, Treg, and Th17 cells during *T. spiralis* infection in pigs. This study is the first to present a detailed kinetic analysis of the immune response in *T. spiralis* infection in pigs. It is important to understand the immunology and pathology of *T. spiralis*.

## Materials and methods

### Parasites and animals

The Chinese *T. spiralis* Henan isolate (ISS534) used in this study was maintained through serial passage in Sprague–Dawley (SD) rats in our laboratory. ML was recovered from the rats infected for more than 30 days post-infection (dpi) by the standard HCL-pepsin artificial digestion method [34]. SD rats and large white pigs were obtained from the Experimental Animal Center of Norman Bethune University of Medical Science (NBUMS), China. All pigs were healthy and were fed a basic diet without any antibiotics. Before the experiment, all pigs underwent routine blood examination, and fecal samples were tested for other parasite eggs by the flotation and sedimentation method. The pigs were kept in a standard pig house in our laboratory under the care of a professional breeder.

All experiments in this study were conducted according to the regulations of the Administration of Affairs Concerning Experimental Animals in China. The procedure of animal experiments was approved by the Institutional Animal Care and Use Committee of Jilin University (Permit No. 20170318).

### Experimental infection

A total of 12 female large white pigs, aged 10 weeks, were randomly divided into 4 groups, in which 3 infected groups were experimentally inoculated with 100 (low-dose group), 1000 (middle-dose group) and 10 000 (high-dose group) ML of *T. spiralis* by oral administration. Another control group was administered 0.9% NaCl solution orally. Serum was collected at 0, 3, 7, 11, 17, 30, 45, and 60 dpi via centrifuging blood samples at 3000 *g* for 30 min at 4 °C, before storage at −80 °C until use. The pigs infected with *T. spiralis* were euthanized at 60 dpi, and 50–100 g samples of six different muscle tissue (tongue, shoulder, flexor, diaphragm, gluteus, and gastrocnemius) were necropsied to calculate the average numbers of larvae per gram (lpg) by digestion method. For the detection of other endoparasites, the stomach, intestine and offal of all pigs were examined at necropsy. The fecal samples were tested for other parasite eggs by the flotation and sedimentation method.

### Flow cytometry

The relative ratios of CD4+ and CD8+ T cells, T cells, B cells, neutrophils, Treg cells, and Th17 cells in peripheral blood were analyzed by flow cytometry. Whole blood samples were obtained from the jugular vein via aspiration as described above. Cells were isolated using red blood cell lysis buffer (Solarbio, Beijing, China) as follows: 200 μL anticoagulation blood was treated with 600 μL red blood cell lysis buffer for 10 min, centrifuged at 1000 *g* for 5 min and discarded from the supernatant. Harvested cells were resuspended in 100 μL cold PBS (phosphate buffered saline, pH 7.2) and then treated for surface and intracellular staining. For surface staining, the following antibodies against surface markers were used according to the manufacturer’s instructions: 2 μL mouse anti-porcine CD3ε-APC (SouthernBiotech, Birmingham, AL, USA), 2 μL mouse anti-porcine CD4-PE (SouthernBiotech), and 2 μL mouse anti-porcine CD8a-FITC (SouthernBiotech) were used to label CD4+ T cells and CD8+ T cells at 4 °C for 30 min in the dark; 2 μL mouse anti-porcine CD3ε-APC and 2 μL mouse anti-porcine CD21-FITC (SouthernBiotech) were used to label T and B cells at 4 °C for 30 min in the dark; 10 μL mouse anti-pig CD14-FITC (Bio-Rad, Hercules, CA, USA), 10 μL mouse anti-pig SWC8 (swine workshop cluster number 8; Bio-Rad), and 1 μL goat anti-mouse IgM-PE (SouthernBiotech) were used to label neutrophils at 4 °C for 30 min in the dark; 2 μL mouse anti-porcine CD4-PE and 2 μL mouse anti-pig CD25-FITC (Bio-Rad) were used for surface labeling of Treg cells at 4 °C for 30 min in the dark; and 2 μL mouse anti-porcine CD3ε-APC and 2 μL mouse anti-porcine CD4-PE were used for surface labeling of Th17 cells at 4 °C for 30 min in the dark. Intracellular staining is required to determine the percentage of Treg and Th17 cells. Next, treatment with 5 μL rat anti-pig FOXP3-APC (eBioscience, San Diego, CA, USA) and 5 μL mouse anti-pig IL-17A-FITC (Mabtech AB, Stockholm, Sweden) were performed at 4 °C for 30 min in the dark following the surface-stained cells fixed and permeabilized with FIX/PERM set (BD Biosciences, San Jose, CA, USA) according to the manufacturer’s instructions. After incubation with antibodies, all stained cells were washed three times with 500 μL cold PBS to remove unbound antibodies. Next, the stained cells were resuspended in 200 μL cold PBS, and the percentage of the stained cells in peripheral blood was examined using a FACSCalibur flow cytometer (BD Biosciences). Data were analyzed using FlowJo software (BD Biosciences).

### Cytokine assay

The levels of Th1 (IL-2), Th2 (IL-4), and Treg (IL-10, TGF-β_1_) cytokines in serum samples at the same time as above were quantified by commercially available ELISA Kits (R&D Systems, Minneapolis, MN, USA) according to the manufacturer’s instructions. Th17 cytokine IL-17A was measured using a commercially available ELISA Kit (Invitrogen, Carlsbad, CA, USA). The concentrations of each cytokine were extrapolated from the standard curve constructed using recombinant porcine cytokines [35].

### Statistical analysis

Statistical analysis was performed using GraphPad Prism 5.0 software for Windows. All data are presented as the mean ± standard error of the means (SEM) of three independent experiments. The two-way ANOVA test was used to analyze the differences between experimental groups. Values of *P < 0.05, **P < 0.01, and ***P < 0.001 were regarded as statistically significant.

## Results

### Larvae densities and distribution of *T. spiralis* infection in pigs

In pigs inoculated with different doses of *T. spiralis* for 60 dpi, larvae densities and distribution in muscle are shown in Table 1. All muscle tissue obtained from infected pigs was larvae-positive. The numbers of larvae in muscle increased in an infective dose-dependent manner with average lpg values of 1.502, 35.947, and 398.811 for pigs infected with 100-ML, 1000-ML, and 10 000-ML doses of *T. spiralis*, respectively. With higher infective doses, the reproductive capacity index (larvae recovered/larvae inoculated, RCI) was higher than that of the low-infective-dose group. In different infective doses, ML were mainly localized to the tongue and diaphragm. All the pigs were in the normal range of leukocytes and eosinophils before the experiment. No gastrointestinal parasites or eggs were found in any of the experimental pigs.

**Table 1:** **Intensity of lpg in muscles of pigs infected with different doses of*T. spiralis***

Location/doses	LPG		
	100	1000	10 000
Tongue	3.339	79.554	782.52
Shoulder	1.001	25.156	316.657
Flexor	0.504	17.37	257.623
Diaphragm	3.528	70.298	726.636
Gluteus	0.266	13.284	179.011
Gastrocnemius	0.371	10.02	130.42
Mean	1.502	35.947	398.811

### Polarization and changes in the proportions of T cell subsets

**Figure 1:**
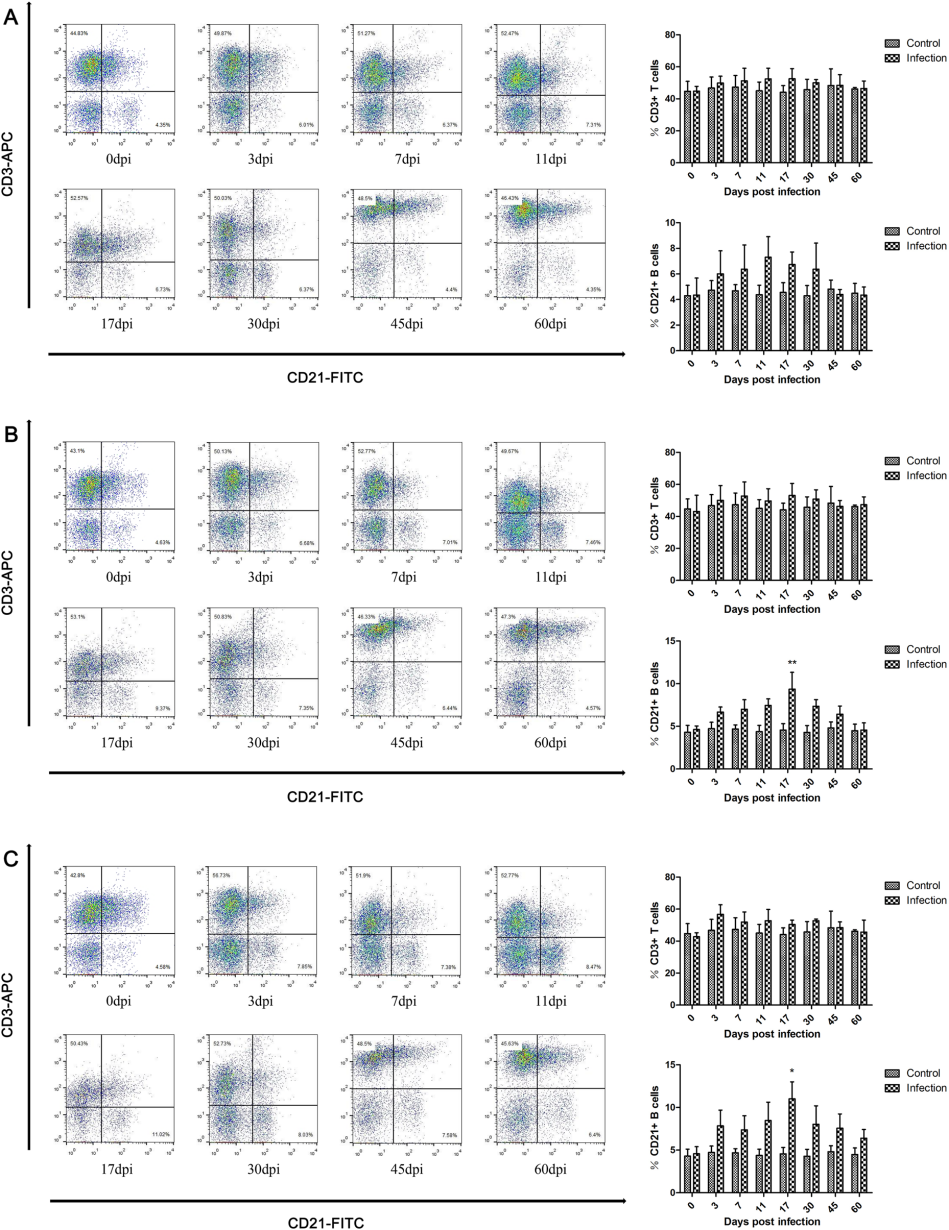
**Dynamics of CD3+ T cells and CD21+ B cells during*T. spiralis*infection.** Quantitative changes in CD3+ T cells and CD21+ B cells in peripheral blood of pigs inoculated with 100 (**A**), 1000 (**B**), and 10 000 (**C**) muscle larvae of *T. spiralis*, as analyzed by flow cytometry. The results shown are the means ± SEMs of three independent pigs per group. *P < 0.05 and **P < 0.01, indicate statistically significant differences compared to the control group.

The proportions of T cell subsets in the peripheral blood of pigs infected with *T. spiralis* were measured. In different infective doses, the total number of CD3+ T cells was slightly elevated in the infection group compared with the control animals after infection but was restored at 45 dpi, as shown in Figure 1.

**Figure 2:**
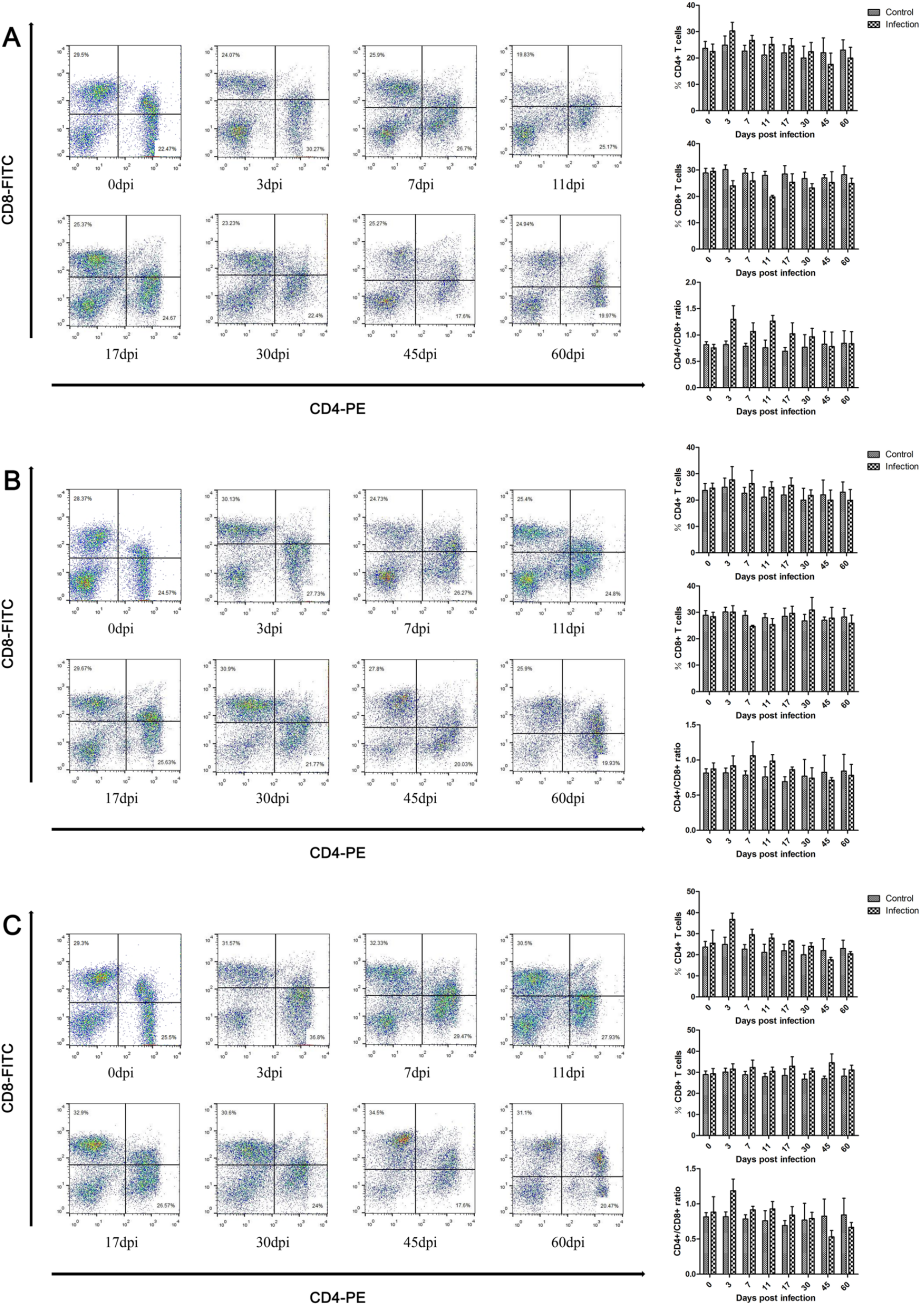
**Dynamics of CD4+ T cells and CD8+ T cells during*T. spiralis*infection.** Populations of CD4+ T cells and CD8+ T cells in peripheral blood of pigs inoculated with 100 (**A**), 1000 (**B**), and 10 000 (**C**) muscle larvae of *T. spiralis*, as analyzed by flow cytometry. The results shown are the means ± SEMs of three independent pigs per group.

The results presented in Figure 2 demonstrate that the proportions of CD4+ T cells peaked at 3 dpi and were lower than the control group after 45 dpi. Instead, CD8+ T cells were reduced in the low-dose group, and the minimum value was observed at 11 dpi (Figure 2A). In the middle-dose group, CD8+ T cells were reduced at 7 dpi, 11 dpi, and 60 dpi (Figure 2B). In the high-dose group, the CD8+ T cell population was increased after infection (Figure 2C). The ratio of CD4+/CD8+ T cells was elevated between 3 dpi and 30 dpi in the low-dose group, peaked at 3 dpi, and was restored at 45 dpi (Figure 2A). In the middle- and high-dose groups, the ratio of CD4+/CD8+ T cells was elevated between 3 dpi and 17 dpi, peaked at 7 dpi and 3 dpi, and decreased at 45 dpi (Figures 2B and C).

**Figure 3:**
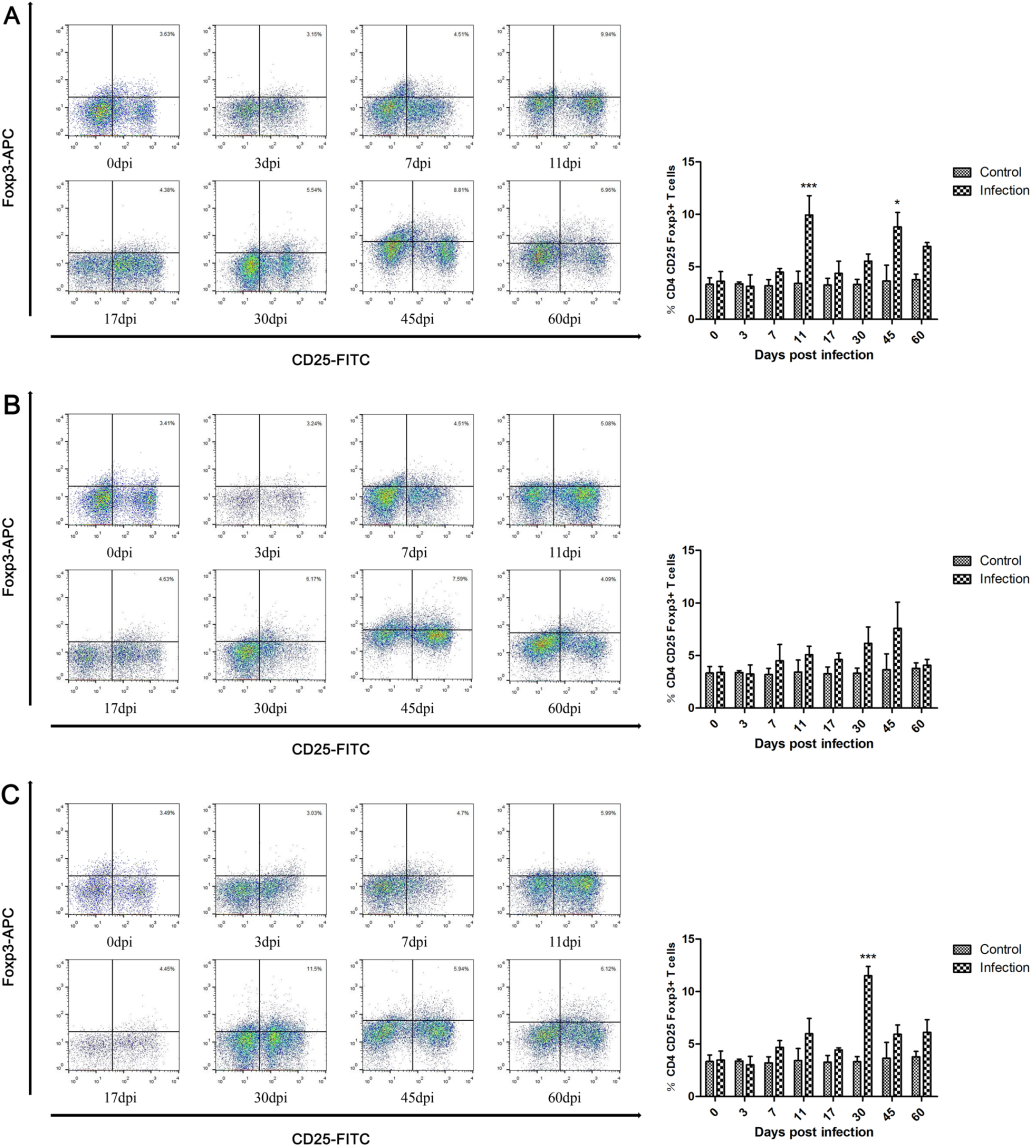
**Dynamics of Treg cells (CD4+CD25+Foxp3+ T cells) during*T. spiralis*infection.** Dynamics of Treg cells in peripheral blood of pigs inoculated with 100 (**A**), 1000 (**B**), and 10 000 (**C**) muscle larvae of *T. spiralis*, as analyzed by flow cytometry. The results shown are the means ± SEMs of three independent pigs per group. *P < 0.05, and ***P < 0.001, indicate statistically significant differences compared to the control group.

A subset of CD4+ T cells, the Treg cells, plays a key role in regulating the host immune response. As shown in Figure 3, at different infective doses, the relative percentages of Treg cells were elevated in the infection group compared with the control animals after 7 dpi and peaked at 11 dpi, 45 dpi, and 30 dpi. The number of Treg cells was significantly increased at 11 dpi and 45 dpi in the low-dose group (Figure 3A) and at 30 dpi in the high-dose group (Figure 3C).

**Figure 4:**
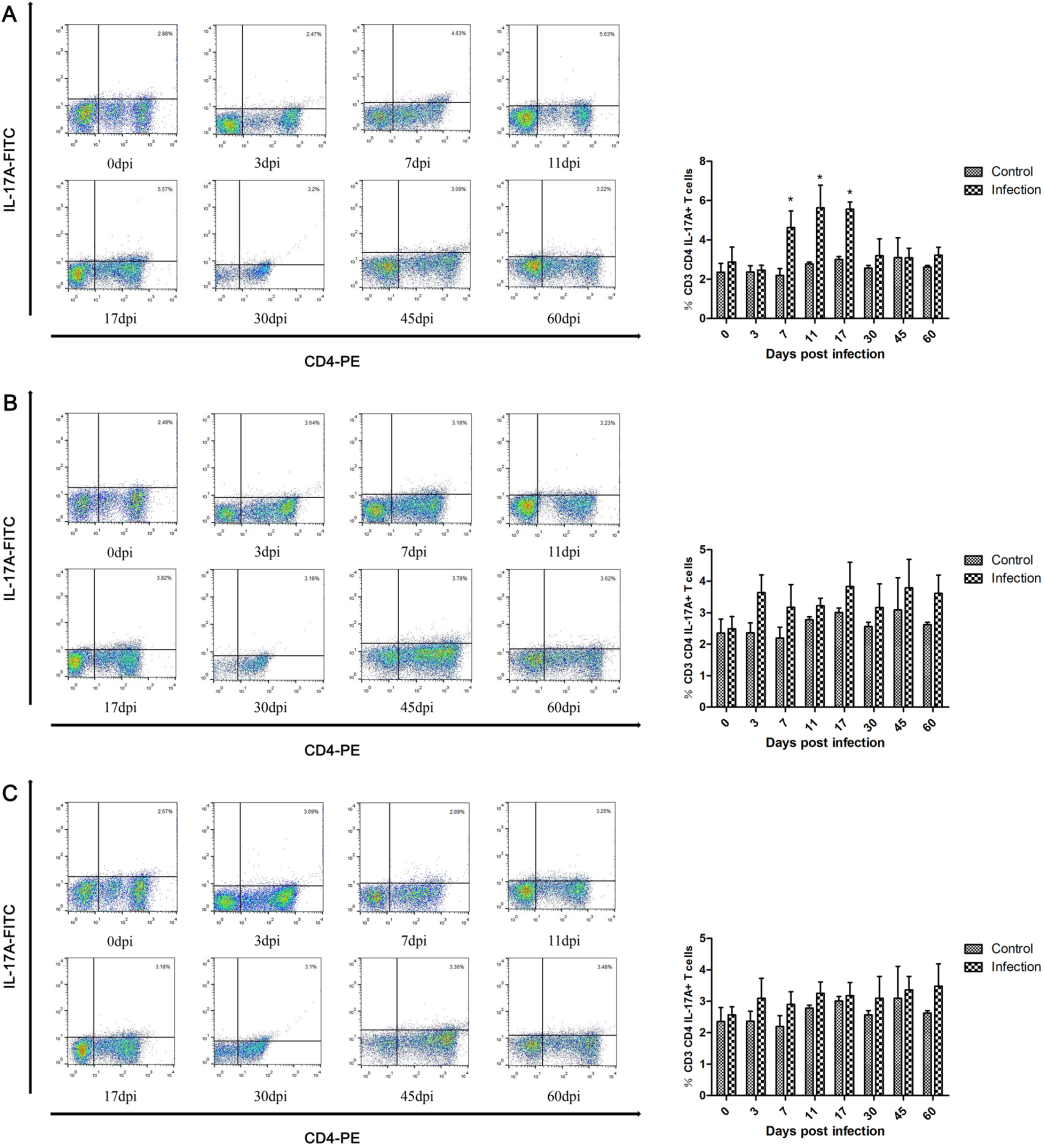
**Dynamics of Th17 cells (CD3+CD4+IL-17A+ T cells) during*T. spiralis*infection.** Dynamics of Th17 cells in peripheral blood of pigs inoculated with 100 (**A**), 1000 (**B**), and 10 000 (**C**) muscle larvae of *T. spiralis*, as analyzed by flow cytometry. The results shown are the means ± SEMs of three independent pigs per group. * P < 0.05 indicates statistically significant differences compared to the control group.

Another subset of CD4 + T cells, the Th17 cells, also participates in the host immune response. The results shown in Figure 4 indicate that the proportion of Th17 cells was significantly increased between 7 dpi and 17 dpi in pigs infected with 100 *T. spiralis*, peaked at 11 dpi and decreased to normal levels as in control animals at 45 dpi (Figure 4A). For the other two infection groups, the proportions of Th17 cells were slightly increased after infection (Figures 4B and C). It is worth noting that the level of increase in Th17 cells was weakened as the infective dose increased.

### Changes in the proportions of B cells

B cells are a major component of the adaptive immune response that can eliminate pathogen infection by producing antibodies and controlling or enhancing T cell function. The results demonstrate that the proportions of B cells were elevated in the low-dose infection group between 3 dpi and 30 dpi, peaked at 11 dpi and were restored at 45 dpi (Figure 1A). In the middle-dose group, B cells were elevated between 3 dpi and 45 dpi, peaked at 17 dpi with significant elevation, and were restored at 60 dpi (Figure 1B). For the high-dose group, the dynamics of B cells were similar to those of the middle-dose group, but the B cell levels continued to increase until 60 dpi and did not return to normal levels (Figure 1C). It should be noted that as the infective dose increased, the production and maintenance time of B cells also increased.

### Changes in the proportions of neutrophil populations

**Figure 5:**
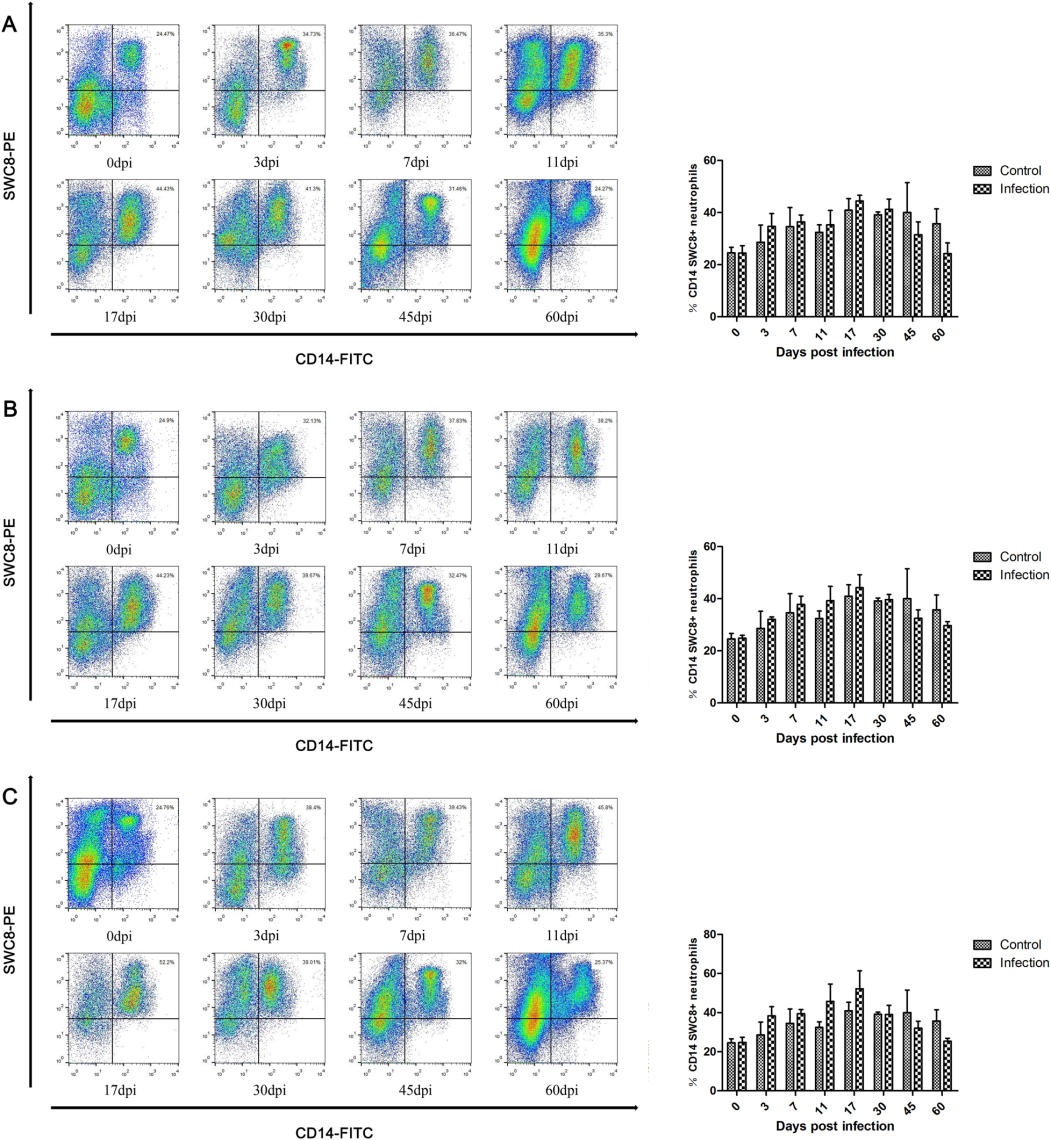
**Dynamics of neutrophils (CD14+ and SWC8+) during *T. spiralis* infection.** Dynamics of neutrophils in peripheral blood of pigs inoculated with 100 (**A**), 1000 (**B**), and 10 000 (**C**) muscle larvae of *T. spiralis*, as analyzed by flow cytometry. The results shown are the means ± SEMs of three independent pigs per group.

Most granulocytes in peripheral blood are neutrophils, which play an important role in nonspecific immune defense by phagocytosis of foreign bodies. As shown in Figure 5, the number of neutrophils in peripheral blood was slightly increased between 3 dpi and 17 dpi after infection with *T. spiralis* and peaked at 17 dpi, and the levels of neutrophils were lower than those in control animals at 45 dpi. The proportions of neutrophils increased with increasing infective dose.

### Serum cytokine expression

**Figure 6:**
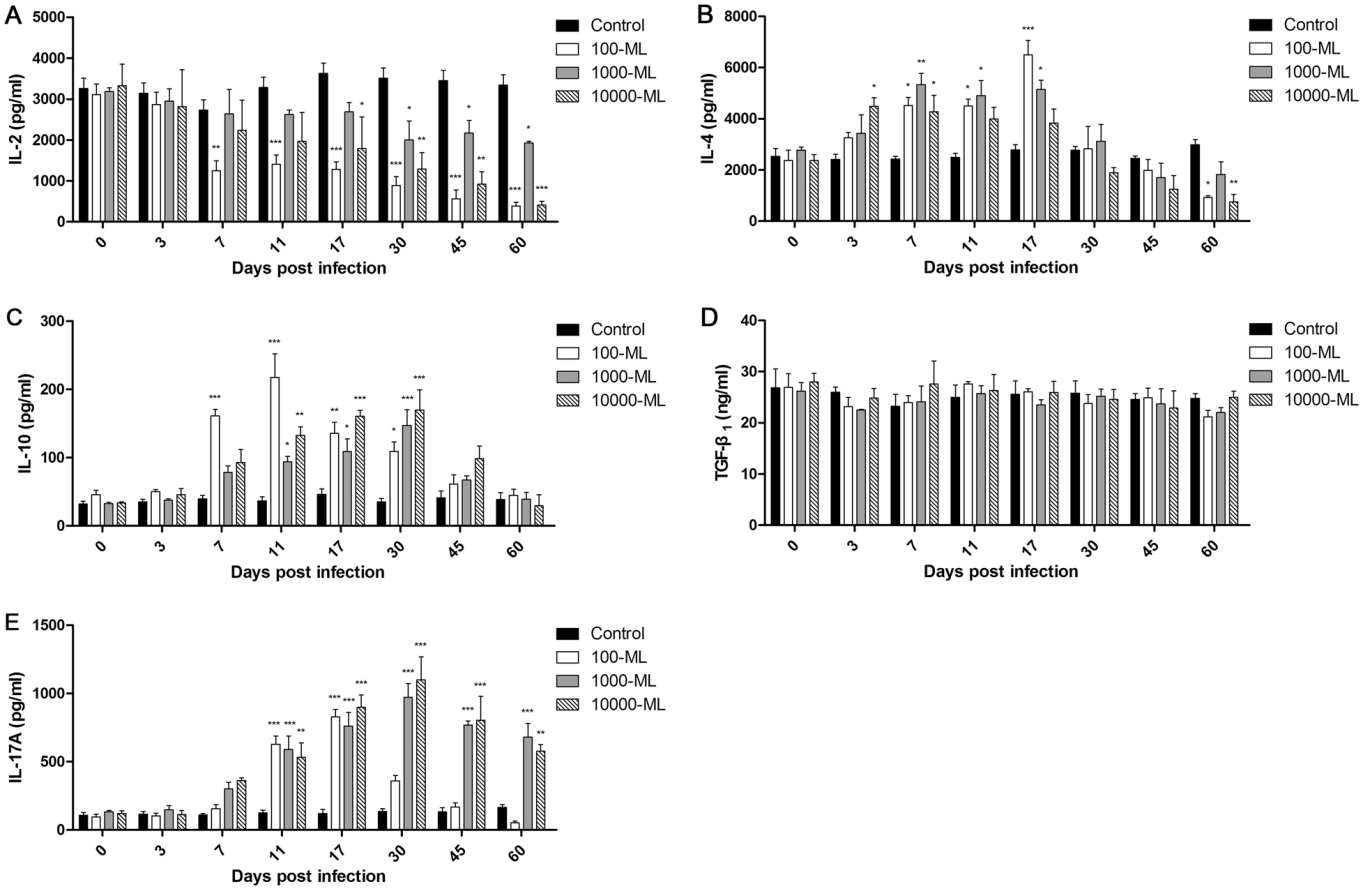
**Cytokine expressions in*T. spiralis*-infected pigs.** The levels of IL-2 (**A**), IL-4 (**B**), IL-10 (**C**), TGF-β_1_ (**D**) and IL-17A (**E**) in serum of pigs inoculated with 100, 1000, and 10 000 muscle larvae of *T. spiralis* were determined by ELISA. The results shown are the means ± SEMs of three independent pigs per group. *P < 0.05, **P < 0.01, and ***P < 0.001 indicate statistically significant differences compared to the control group.

To investigate CD4+ T cell differentiation, the expression levels of the cytokines associated with Th1, Th2, Treg, and Th17 immune responses were measured in serum by ELISA. The results shown in Figure 6 indicate that at the different infective doses, the levels of IL-2 decreased compared with healthy animals after infection and reached a minimum at 60 dpi. At the same time, the earliest significant reduction appeared at 7 dpi in the low-dose infection group (Figure 6A). The levels of IL-4 were significantly elevated between 3 dpi and 17 dpi and peaked at 17 dpi, 7 dpi, and 3 dpi in the low-dose, middle-dose, and high-dose infection groups, respectively. In addition, IL-4 levels were lower than in healthy animals at 45 dpi, except for the high-dose infection group, which was decreased at 30 dpi (Figure 6B). The levels of IL-10 were significantly elevated between 7 dpi and 30 dpi, were restored at 60 dpi and peaked at 11 dpi, 30 dpi, and 30 dpi in the low-dose, middle-dose, and high-dose infection groups, respectively (Figure 6C). There was no significant change in the expression of TGF-β_1_ in pigs infected with *T. spiralis* (Figure 6D). The levels of IL-17A were significantly increased beginning at 11 dpi in the low-dose, middle-dose, and high-dose infection groups, which peaked at 17 dpi, 30 dpi, and 30 dpi, respectively. However, the IL-17A levels were restored at 45 dpi and were lower than those of healthy animals at 60 dpi in the low-dose infection group (Figure 6E).

## Discussion

Characterizing the immune response processes is key to understand host–parasite interactions. *T. spiralis* ML could live in the host for various years, although the host immune system is activated to accelerate worm expulsion from the intestine. One explanation for this phenomenon is that nurse cell formation in the ML stage protected the parasite from specific antibody attack appearing after the fourth week of infection [12]. Another possibility is that the parasite could moderate the host immune response to facilitate parasite survival using secretory products and surface antigens [12, 17]. In mice, the host immune response varies at different stages of *T. spiralis* infection [18]. However, the immune responses of mice and pigs are different [36]. This difference might prevent many potential candidates for vaccines and diagnostic targets with high protective efficacy and diagnostic efficacy in murine models from being applicable to pigs. In addition, the dynamics of immune cells and cytokines in pigs infected with *T. spiralis* have not been determined. Therefore, in this study, we characterized for the first time the fundamental immunology of pigs infected with *T. spiralis*.

It was reported that ML can be detected in mice infected with doses as low as 5 L1 *T. spiralis* [37]. However, the body weight of pigs is significantly higher than that of mice; meanwhile, the pigs infected with 25 and 50 ML exhibited few ML in the diaphragm and tongue of all animals (our unpublished data). Therefore, we increased the lowest infective dose to 100 ML, which was lower than that in a previous study, which employed 200 ML as the lowest infection dose in pigs [38]. In this study, the results of artificial digestion of infected swine carcasses showed that all the infection groups were larvae-positive, even in the 100-ML infection groups. The intensity of recovered ML in muscle tissue was positively correlated with inoculated doses, and the RCI increased with inoculated doses. This suggested that the inoculated doses influence worm burden, which is consistent with the results of a previous study investigating horses and rats, even in long-term infection [39, 40]. It is natural that increasing the infection dose increases ML burden due to increase of NBL. The lower RCI in the low-infective-dose group may be due to the presence of significant Th2 and Treg immune responses, which suppress the Th1 immune response and promote worm expulsion [12, 20]. Meanwhile, a significant Th17 immune response in the low-infective-dose group participated in worm expulsion at the intestinal stage [26].

The cellular immune response plays a major role in parasite infection, affecting such cells as T cells, B cells, neutrophils, eosinophils, macrophages, mast cells, and goblet cells. T cells can perform two different functions by regulating the immune response: the worm expulsion and promoting worm survival. At the intestinal stage, the mechanisms of adult worm and NBL expulsion are mainly divided into the following points: the inflammatory response regulated by T helper cells plays a key role in the process of worm expulsion; a direct immune response to the stage-specific antigens; through the immunological mechanisms to worm expulsion [12]. NBL survival through stage-specific antigens and modifications of surface proteins, which cannot be bound to early specific antibodies for adult worms [41]. In addition, the lymphocytotoxic factors were released in NBL stage [12]. Anatomic seclusion was important to ML survival, and for the ML continuously releasing antigens to stimulate the host immune response in the muscle stage, the nurse cell protecting it from antibody and effector cell attack. To persistently survive in the host, the ML could modulate and suppress the host immune response [12]. In the muscle stage, the pathology of muscle mainly was damage in the myocardium, lungs and central nervous system caused by enriched eosinophils in the blood and tissues of the infected hosts [42].

The present study observed that the proportion of T cells was slightly elevated after infection and returned to basal levels sat later stages of infection. This finding was probably due to the rise of CD4+ T cells in all infection groups and CD8+ T cells in the high-dose group. CD4+ T cells are generated in the intestinal phase of *T. spiralis* after 2–4 dpi and play a key role in the immune response [43, 44]. Our results showed that CD4+ T cells increased after infection and peaked at 3 dpi, which was similar to the results obtained by previous studies [43, 44]. Diarrhea was observed in humans at the intestinal phase of the infection due to inflammation caused by adult worms migrating to Peyer’s patches and development and continuous antigen stimulation exposure to the host [45]. The immune response was important to adult worm expulsion at the intestinal phase, which was dependent on CD4+ T cells [46]. However, the proportions of CD4+ T cells were lower in the infected group after 45 dpi. It may be that the immunomodulation strategy induced by Treg immune response in *T. spiralis* infection, which is able to tame the Th1, Th2, and Th17 immune responses [17]. Consequently, the proportions of CD4+ T cells were reduced in the muscle stage. CD8+ T cells play a key role in worm expulsion by the subset of CD8+ cytotoxic T cells (Tc), which could activate mononuclear phagocytes to kill parasites [47]. In the lower and middle infection groups, CD8+ T cells were suppressed by infection with *T. spiralis*, and this suppression was important to parasite survival at the intestinal phase. However, the number of CD8+ T cells was increased in the highest-dose infection group after infection, possibly because large numbers of worms cause severe damage and inflammation in the intestinal and muscle tissues, which triggers an expulsion worm response. It has been reported that both CD4+ and CD8+ T cells were increased in infected mice with 500 ML, and the levels of CD8+ T cells were increased higher than CD4+ T cells and with a declining ratio of CD4+/CD8+ T cells [43]. In our study, the ratio of CD4+/CD8+ T cells was elevated at the intestinal phase, which meant that the most serious suppression of the immune response occurred during the intestinal phase of pigs infected with *T. spiralis*. The results indicated that the immune response process of pigs and mice was different during infection with *T. spiralis*.

Previous studies demonstrated that the immune system of rodents and humans infected with *T. spiralis* is driven to a less dangerous response, in which Th2 was predominant with a short Th1 immune response [10, 12]. The Th2 immune response was induced in parasitic infections to inhibit Th1 activation. In humans, the Th2 immune response persists in chronic infection of *T. spiralis*, which is important to the protective immune response [11]. IL-4 is involved in protective Th2 immune responses and promotes the production of IgE, which is a Th2-associated humoral response [48, 49]. The aim of the Th1-type immune response is to eliminate the parasite. However, this response damages the host, which favors the survival of the parasite [20]. The Th1 type cytokine IL-12 delays worm expulsion and increases worm burden in an IFN**γ**-independent manner [50]. The Th2 immune response induced by *T. spiralis* infection plays a key role in intestinal immunity for adult worm expulsion [12]. In our study, the inhibition of the Th1 immune response was accompanied by an elevation of the Th2 immune response at 7 dpi, which promoted adult worm expulsion at the intestinal stage. The results indicated that down regulation of the Th1 immune response may be due to Th2 immune response activation. Although the worm expulsion existed at the intestinal phase of the infection, the parasite could modulate and suppress the immune response by secreting antigens to escape from the host immune response [12, 51]. The parasite stimulates the production of Treg cells to homeostatic inflammation responses of hosts [52]. At the early stages of muscle infection, Treg cytokine IL-10 is able to limit local and regional inflammation, and TGF-β also regulates local inflammation response in infected muscle, which facilitates NBL and ML survival. Conversely, depletion of Treg cells results in exaggerated Th2 responses [53, 54]. Meanwhile, immunomodulation of *T. spiralis* infection has a therapeutic effect on chronic autoimmune diseases, such as colitis and encephalomyelitis (EAE) [55, 56]. In this study, the activation of the Treg immune response begins at 7 dpi and maintains high levels when encapsulation is formed, which is important to control the inflammatory response and NBL and ML survival at the intestinal and muscle stages. The above theory was confirmed by our results indicating that the levels of Th2 and Treg cells were significantly increased after pigs were infected with *T. spiralis*, and Th1 cell levels declined, which was different from the results obtained in rodents and humans with a short Th1 immune response [10, 12]. Interestingly, the immune responses regulated in *T. spiralis* infection had a focus on the NBL production phase. It may be that the worms have different developmental stages and born larvae at the intestinal phase, releasing stage-specific antigens and surface antigens that are frequently exposed to hosts, inducing the immune response. Immunomodulation was decreased when nurse cells formed. The most obvious immune regulation occurs in low-dose infection groups, which cannot be explained at present and warrants further investigation.

Other inflammatory cells, such as neutrophils and Th17 cells, were increased in the peripheral blood of infected pigs. Neutrophils play a key role in the innate immune system responses, which are able to phagocytose foreign matter [29]. The functions of neutrophils were to promote inflammation and fight infections. In the present study, neutrophils increased after infection and peaked at 17 dpi, which may be associated with expulsion worms by killing NBL in antibody-dependent cell-mediated cytotoxicity (ADCC) systems [57, 58]. It was reported that the levels of IL-17A, which is involved in the activation of Th17 cells, were increased during the intestinal phase of *T. spiralis* infection, IL-17A is a pro-inflammatory mediator and contributes to jejunal muscle contractility that may contribute to parasite expulsion from the gut [26]. IL-17A promotes the increased number of neutrophils in the airways [59]. Our results indicated a similar increase in pigs infected with *T. spiralis*. However, the levels of IL-17A were significantly higher than the proportion of the Th17 cells, and the changes of Th17 cells in lower infection doses were significant than higher infection doses. The mechanism underlying the regulation of Th17 cells warrants further investigation.

Humoral responses were induced by *T. spiralis* in the immune response of hosts [17]. Antibodies were the key component of humoral immunity produced by B cells. The IgG antibodies can kill the NBL by induced eosinophils and ADCC [43]. A previous study demonstrated that the number of B cells significantly increased at 15 dpi in mice infected with *T. spiralis* [43]. Our results showed that B cells were significantly increased in the middle-dose and high-dose groups at 17 dpi, which was similar to the results of a previous study in mice [43]. Meanwhile, as the infection dose increased, the production and maintenance time of B cells also increased. This finding may be attributable to high doses of infection with large amounts of secretory antigens and surface antigens exposure for the host to continuously stimulate the proliferation of B cells.

In conclusion, based on these findings, *T. spiralis* achieves successful parasitism by regulating the immune response of the host. Our results confirmed that pigs infected with *T. spiralis* predominantly induced Th2 and Treg immune responses, which suppress the Th1 immune response. These observations provide a valuable information for further study of the immunology and parasitology between pigs and parasites and are important for the diagnosis and treatment of *T. spiralis* infection.

## Data Availability

All data generated or analyzed during this study are included in this published article.

## Abbreviations

ELISA: enzyme-linked immunosorbent assay
ANOVA: analysis of variance
ML: muscle larvae
Ad: adult worm
NBL: new born larvae
dpi: days post-infection
lpg: larvae per gram
IL: interleukin
TGF-β: transforming growth factor-beta
ADCC: antibody-dependent cell-mediated cytotoxicity
Tc: cytotoxic T cells
APCs: antigen-presenting cells
RCI: reproductive capacity index
SWC8: swine workshop cluster number 8
